# Anti-inflammatory and antinociceptive effects of phonophoresis in animal models: a randomized experimental study

**DOI:** 10.1590/1414-431X20187773

**Published:** 2019-01-24

**Authors:** L.C.P. Cardoso, N.B. Pinto, M.E.P. Nobre, M.R. Silva, G.M. Pires, M.J.P. Lopes, G.S.B. Viana, L.M.R. Rodrigues

**Affiliations:** 1 Programa de Pós-Graduação Stricto Sensu em Ciências da Saúde, Faculdade de Medicina do ABC, Santo André, SP, Brasil; 2 Unidade Acadêmica Ciências da Vida, Universidade Federal de Campina Grande, Cajazeiras, PB, Brasil; 3 Faculdade de Medicina, Universidade Federal de Cariri, Barbalha, CE, Brasil; 4 Departamento de Farmacologia, Universidade Federal do Ceará, Fortaleza, CE, Brasil; 5 Departamento de Ciências da Computação, Inteligência e Processamento de Imagens, Universidade Federal Rural de Pernambuco, Serra Talhada, PE, Brasil; 6 Laboratório de Fisiologia, Faculdade de Medicina Estácio de Juazeiro do Norte, Juazeiro do Norte, CE, Brasil

**Keywords:** Phonophoresis, Ultrasonic therapy, Inflammation, Nociception, Physiotherapy

## Abstract

The aim of this study was to evaluate the therapeutic effects of ultrasound (US)-mediated phonophoresis alone or in association with diclofenac diethylammonium (DCF) administered topically in animal models of inflammation. A pre-clinical, prospective, and randomized experimental study of quantitative and qualitative nature was carried out. Phonophoresis was performed using a therapeutic ultrasound apparatus in two distinct models of acute inflammation. Edema was induced by an intraplantar injection of carrageenan and measured by plethysmography. The Hargreaves test was used to evaluate the antinociceptive activity and investigate the action of phonophoresis on tumor necrosis factor (TNF)-α production. A histological analysis with hematoxylin-eosin was used to evaluate tissue repair, and the expression of COX-2 was determined by immunohistochemical analysis. At the peak of inflammatory activity (3 h), treatment with US, US+DCF, and DCF significantly reduced edema formation compared to the control group. Treatment with US+DCF was more effective than treatment with US alone at both analyzed times. In the analysis of the antinociceptive activity, the treatments significantly increased the latency time in response to the thermal stimulus. Histopathological analysis revealed a reduction of the inflammatory infiltrates and immunohistochemistry demonstrated that the association was effective in reducing COX-2 expression compared to the control group. The association of DCF with US produced anti-inflammatory and antinociceptive effects in rat models of inflammation, which may be associated with inhibition of COX-2 and TNF-α production.

## Introduction

The skin is the largest organ of the human body. Its main function is to coat and protect tissues, limiting the interaction between the internal structures and the external environment. The use of the transdermal route for therapeutic purposes has numerous benefits, including maintaining stable plasma levels of drugs, avoiding gastrointestinal degradation, and limiting hepatic effects due to the absence of first-pass metabolism (1–3).

Phonophoresis can be described as a non-invasive technique that uses piezoelectric potential by converting electrical energy into mechanical energy. In practice, this effect is obtained from the production of high-frequency oscillation sound waves, directed by the therapeutic ultrasound apparatus (US). This action provides a controlled and safe way to potentiate the transdermal absorption of a wide variety of ionizable drugs without causing significant discomfort. Thus, the action of many drugs, such as anti-inflammatories and analgesics that are administered to the outer layer of the skin, is potentiated as they penetrate in the deeper underlying tissue (4–7).

Previous studies have suggested that the mechanism underlying the deposition of the drug with this process is cavitation. In cavitation, the action of the US results in the formation of gaseous microbubbles in the outer layer of the skin that can rupture violently, favoring the penetration of the drug. Additionally, cavitation causes changes in the organization of the lipids in the stratum corneum, increasing its permeability, which contributes to several physiological and therapeutic effects, including activation of blood flow, improvement of tissue nutrition, oxygenation, and metabolism, as well as rearrangement and regeneration of collagen fibers in connective tissues (8–10). Therefore, due to its promising therapeutic effects, phonophoresis has been widely employed in clinical physiotherapy for the treatment of musculoskeletal disorders, especially in the rehabilitation of patients with acute and chronic osteomioarticular lesions, bone inflammation, pain, and edema (11,7).

Currently, there are some studies demonstrating the effects of the association between drugs and phonophoresis at different conditions of exposure to ultrasonic waves (e.g., intensity, frequency, duration, and continuity) (12). However, the effectiveness of this technique in improving the therapeutic effect of drugs remains to be better investigated (4).

Therefore, the objective of this work was to evaluate the therapeutic effects of the ultrasound-mediated phonophoresis alone or in association with diclofenac diethylammonium topically administered in experimental models of inflammation.

## Material and Methods

### Design

This was a preclinical, prospective, and randomized experimental study of quantitative and qualitative nature.

### Animals

This study was carried out using male Wistar rats weighing 180–250 g, divided into 5–6 groups of 6 animals in two different protocols (total n=66 animals) as described below. The animals were housed in boxes at 24±2°C under a 12/12 h light/dark cycle with free access to the standard diet (Purina Chow) and potable water. They were deprived of food (but not water) for 8 h prior to the experiments. The experiments were carried out according to the guidelines of Animal Research: Reporting of *in vivo* Experiments (ARRIVE), the UK Animals (1986), and associated guidelines, and the Committee on Ethics in the Use of Animals (CEUA) of the Faculdade de Medicina Estácio do Juazeiro do Norte (FMJ), which approved this study (protocol No. 2015.1-007).

### Intervention

An acute inflammatory process was induced using the paw edema model. Briefly, 0.1 mL carrageenan (1% w/v) in saline was administered in the subplantar area of the right hind paw. Diclofenac diethylammonium (DCF) at 10 mg/g was administered topically in the form of a gel before the application of phonophoresis. This method was performed using a microcontrolled ultrasound device (Sonopulse Ibramed, Brazil), with the following specifications: ultrasound frequency of 1.0 Mhz; adjustable intensity of 1.0 W/cm^2^ with maximum output power of 3.5 W; effective radiation area of 5 cm^2^ (ultrasound and transducer), mode of emission of pulsed ultrasound (the most indicated for acute processes (1); pulse ratio=1: 5–20%, frequency of 100 Mhz), method of coupling: direct and steady with oscillatory movements, for 1 min, in a single application. The time of 1 min was determined from pilot tests in which more representative results were obtained when the application was performed for 1 min, compared with the time of 30 s. The animals were placed on a table in dorsal decubitus, the paw was immobilized, and the ultrasound was applied perpendicularly (90°) on the shaved skin in the right plantar fascia.

To evaluate the effects of phonophoresis alone on inflammation and nociception, two different protocols were used. Protocol 1 was used to evaluate the anti-inflammatory and antinociceptive effects of phonophoresis in the paw edema test. Protocol 2 was used to evaluate the anti-inflammatory and antinociceptive effects of the phonophoresis associated with 1 mg/kg pentoxifylline (PENTOX 1) in paw edema tests, as described in Tables 1 and 2.

**Table 1.** Protocol 1 for evaluation of the anti-inflammatory and antinociceptive effects of phonophoresis in the paw edema test.

GROUP	DRUG/DOSE
1) CONT: Negative control, carrageenan injury without therapy.	No therapy
2) IND: Positive control, injury	Indomethacin (20 mg/kg, *po*)
3) US: lesion	Phonophoresis with aqueous commercial gel (1 mHz, pulsed, 1.0 w/cm^2^ ultrasound for 1 min). Topical route.
4) DCF: lesion	Diclofenac diethylammonium (10 mg/g). Topical route.
5) US+DCF: lesion	Phonophoresis with aqueous commercial gel (1 mHz, pulsed, 1.0 w/cm^2^ ultrasound for 1 min). Topical route.

Cont: Control; IND: indomethacin; US: ultrasound; DCF: diclofenac diethylammonium.

**Table 2.** Protocol 2 for evaluation of the anti-inflammatory and antinociceptive effect of phonophoresis associated with pentoxifylline in paw edema tests.

GROUP	DRUG/DOSE
1) CONT: Negative control, carrageenan injury without therapy	No therapy
2) IND: Positive control, injury	Indomethacin (20 mg/kg, *po*)
3) PENTOX 1: Injury	Pentoxifylline at the dose of 1 mg/kg (*ip*)
4) PENTOX 1 + US: Injury	Pentoxifylline at the dose of 1 mg/kg (*ip*) + US (US 1 mHz, pulsed, 1.0 w/cm^2^ per 1 min)
5) PENTOX 1 + US + DCF: Injury	Pentoxifylline at the dose of 1 mg/kg (*ip*) + US (US 1 mHz, pulsed, 1.0 w/cm^2^ per 1 min) + diclofenac diethylammonium (10 mg/g). Topical route.
6) PENTOX 1 + DCF: Injury	Pentoxifylline at the dose of 1 mg/kg (*ip*) + US (US 1 mHz, pulsed, 1.0 w/cm^2^ per 1 min) + diclofenac diethylammonium (10 mg/g). Topical route.

Cont: Control; IND: indomethacin; PENTOX: pentoxifylline; US: ultrasound; DCF: diclofenac diethylammonium.

### Outcome measures

The analyses were performed from randomized trials in which the acute edema of the hind paw was measured after administration of carrageenan, a phlogistic agent commonly used in models of acute inflammation. The treatments were performed 1 h prior to carrageenan injection, as follows: indomethacin was administered orally (*po*) and pentoxifylline intraperitoneally (*ip*) as detailed in Tables 1 and 2.

#### Primary outcome

Edema was defined as the difference between the volumes of the right and left paws from the first to the fourth hour after administration of carrageenan and the basal levels of the edema were determined before the application of the stimulus. All measurements were recorded using a plethysmograph (Ugo Basile, Italy).

#### Secondary outcome

The Hargreaves test was used to evaluate the antinociceptive activity. Briefly, a radiant source of infrared light was administered to the central region of the hind paw of rats, causing warming. This test measures the effect of the drug on a peripherally mediated response to thermal stimulation in the rat (spontaneous movement). The nociceptive behavior was defined as heat sensitivity (thermal hypernociception) after application of a light ray, and the nociceptive response was determined according to the latency time until paw withdrawal. Of note, this response is usually inhibited by cyclooxygenase (COX) inhibitors (13). The oral treatments were performed 1 h before the intraplantar injection of carrageenan. The treatments (DCF and US) were performed topically immediately after application of carrageenan and the pain response was evaluated 1 h after the administration of this inflammatory agent. In this test, a group of animals that received no treatment or inflammatory stimulus was used as control. The paw withdrawal latency was quantified after triggering the infrared light using a time counter system, which ceases with the paw withdrawal response.

After intervention, the animals were intraperitoneally anesthetized with thiopental sodium (50 mg/kg). Then, the tissues were removed from the plantar region of the right hind paw. While still anesthetized, the animals were euthanized by cervical dislocation. After collection, the samples were placed in cassettes with filter paper and dipped in 10% non-buffered formaldehyde for 24 h. Histological sections were embedded in paraffin and subsequently stained with hematoxylin-eosin (HE). The histological sections were analyzed microscopically for evaluation of tissue repair.

The slides were fixed in 10% formaldehyde for 24 h, treated with 70% alcohol solution and embedded in paraffin. Histological sections of 5 μm thickness were placed on glass slides and prepared for immunohistochemistry for COX-2. Three slides of three different animals were made for each group and kept in a histological oven at 58°C for 10 min before the beginning of the experiments.

### Data analysis

The results are reported as means±SE. For multiple comparison of parameters, one-way ANOVA followed by Student-Newman-Keuls or Tukey's test as *post hoc* tests were used. Analyses were performed using the Graph Pad Prism 5.0 software (USA), with a confidence interval of 95%. Differences with a P≤0.05 were considered significant. Histological analyses were performed using a BX-41 optical microscope (Olympus, Japan) with a coupled camera (Olympus) and photomicrographs were analyzed using the ImageJ software (NIH, USA). These data were analyzed based on the technique of segmentation to select in the images the parts corresponding to the nuclei of cells corresponding to leukocyte infiltrates in the dermis, through the relative frequency.

## Results

### Measurement of edema and inflammatory activity

**Figure 1.**
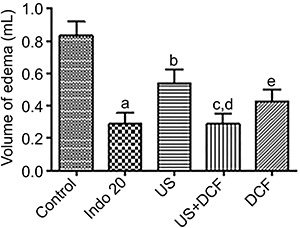
Evaluation of acute treatment on the volume of paw edema 3 h after carrageenan administration in rats treated with ultrasound (US), ultrasound associated with diclofenac diethylammonium (US+DCF), and diclofenac diethylammonium alone (DCF). Data are reported as mean±SE. Indomethacin 20 mg/kg (*po*) was used as reference drug. ^a^P<0.001, ^b^P<0.05, ^c^P<0.001, ^e^P<0.01 *vs* control; ^d^P<0.05 *vs* US (ANOVA).

In the experimental model of paw edema induced by carrageenan, the peak of inflammatory activity occurs three hours after the challenge and is orchestrated by prostaglandins that increase vascular permeability (14). In the present study, treatment with US (0.54±0.09), US+DCF (0.29±0.07), and DCF (0.43±0.00) significantly decreased (35, 65, and 48%, respectively) the paw edema induced by carrageenan compared to the control group (0.83±0.09). In addition, treatment with US+DCF (P<0.001) was significantly more effective than treatment with US alone (P<0.05) (Figure 1) and presented an effectiveness that was comparable to that of the positive control (INDO 20). These results indicated that the treatments presented antiedematogenic effects and that phonophoresis improved the antiedematogenic effect of diclofenac in this experimental model.

**Figure 2.**
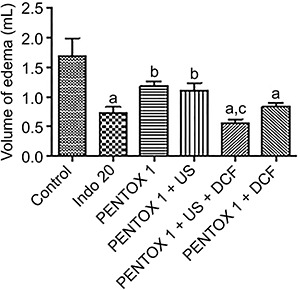
Means±SE of the volume of paw edema 3 h after administration of carrageenan in diclofenac (DCF) pre-treated rats with or without ultrasound (US+DCF) and pentoxifylline (PENTOX 1). Indomethacin 20 mg/kg (*po*) (INDO 20) was used as reference drug. ^a^P<0.05, ^b^P<0.001 vs control; ^c^P<0.05 *vs* PENTOX 1, PENTOX 1+US, and PENTOX 1+DCF (ANOVA).

The groups described in Table 2 were used to evaluate the effect of pentoxifylline associated with US and DCF on carrageenan-induced paw edema. The use of pentoxifylline aimed to determine the action of phonophoresis on TNF-α production as well as to evaluate the synergism between the treatments. An acute treatment with diclofenac associated or not with ultrasound and pentoxifylline significantly reduced the volume of paw edema 3 h after the challenge. Animals treated with PENTOX 1+US+DCF (0.560±0.06), PENTOX 1 (1.187±0.08), PENTOX 1+US (1.102±0.14), and PENTOX 1+DCF (0.843±0.057) presented significantly lower values of edema (67, 30, 35, and 50%, respectively) compared to the negative control. Indomethacin (INDO) was used as a positive control and it decreased the edema volume by 57% (Figure 2).

### Assessment of antinociceptive activity

**Figure 3.**
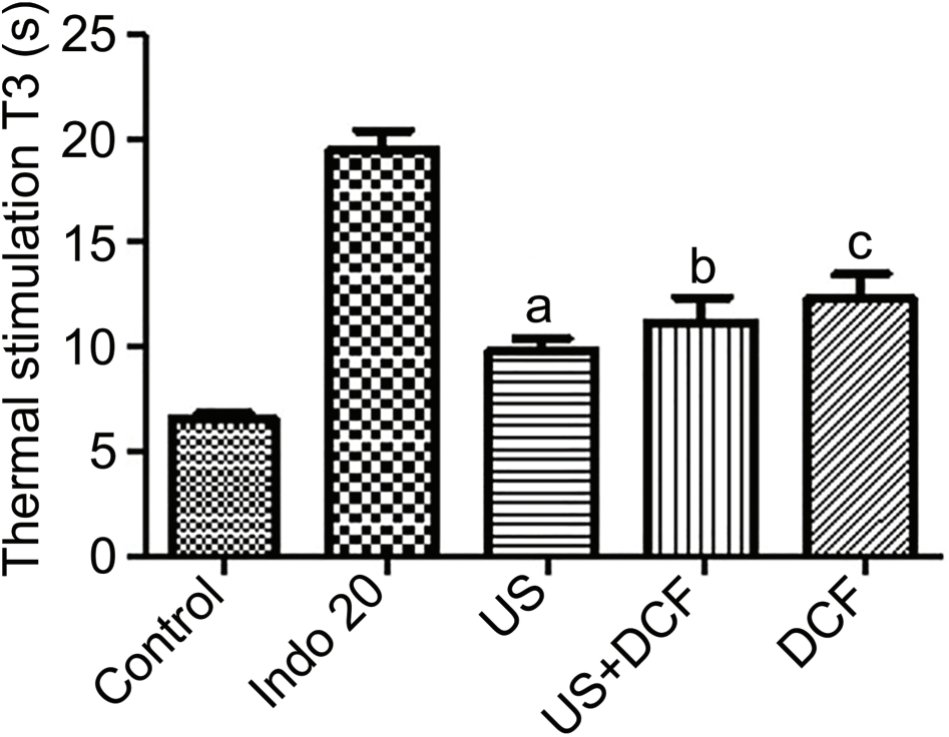
Evaluation of acute treatment with diclofenac (DCF) or ultrasound-associated DCF (US+DCF), and US on thermal nociception in cutaneous hyperalgesia (Hargreaves's test) in the third hour (T3). Data are reported as mean±SE. Indomethacin 20 mg/kg (*po*) (INDO 20) was used as reference drug. ^a^P<0.05; ^b^P<0.01; ^c^P<0.001 *vs* control (ANOVA).

The groups treated with US (9.9±0.6), US+DCF (11.2±1.2), or DCF (12.3±1.2) significantly increased (P<0.05, 0.01, and 0.001, respectively) the latency time to the heat stimulus compared to the control group (6.5±0.3) in the Hargreaves test, indicating that these treatments have comparable antinociceptive effects on cutaneous hyperalgesia. Indomethacin (19.5±0.9), which was used as a standard control drug, increased the latency time to thermal stimulation by 3-fold (Figure 3).

### Histopathological analysis

**Figure 4.**
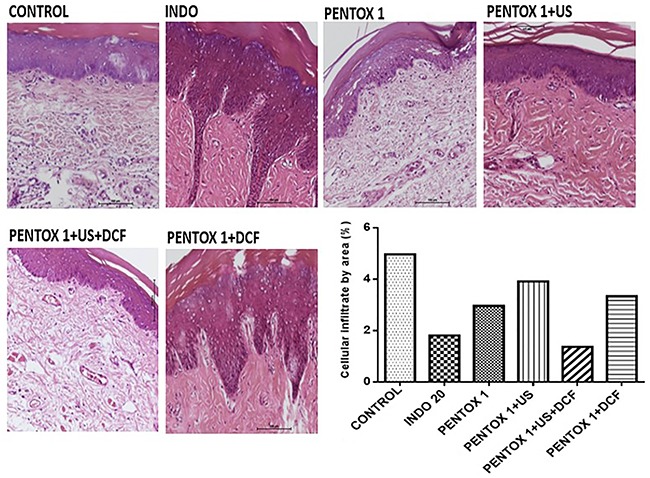
Photomicrographs representative of histopathological analysis (100×, bar=200 μm) of tissues from the right paw of rats submitted to the carrageenan-induced paw edema model, stained with hematoxylin-eosin. The images demonstrate cell infiltrates for the Control and groups treated with 20 mg/kg indomethacin, *po* (INDO 20), 1 mg pentoxifylline (PENTOX 1), 1 mg pentoxifylline associated with ultrasound (PENTOX 1+US), 1 mg pentoxifylline associated with ultrasound, and 10 mg diclofenac (PENTOX 1+US+DCF), 1 mg pentoxifylline associated with 10 mg diclofenac (PENTOX 1+DCF). Percent of cellular infiltrate analyzed by the ImageJ program is shown in the graph. Data are reported as means.

Microscopic analysis of tissue sections obtained from the paws of animals challenged with carrageenan revealed the presence of abundant leukocyte infiltration in the dermis of control animals (4.97%). These inflammatory infiltrates were reduced in all treated groups. However, the PENTOX 1+US+DCF group presented the lowest percentage of cell infiltrates (1.36%) compared to the other groups, including the group treated with indomethacin (1.81%), suggesting that, when associated, these treatments show synergism (Figure 4).

### Immunohistochemical analysis

Carrageenan is an inflammatory agent that is widely employed in experimental models of edema and hyperalgesia, especially in studies involving non-steroidal anti-inflammatory drugs (NSAIDs). It is known that peripheral inflammation involves an increase in the expression of COX-2, which catalyzes the synthesis of prostaglandins in the central nervous system, contributing to allodynia and hyperalgesia (15). In this process, carrageenan also induces neutrophil migration, which is stimulated by the release of TNF-α.

**Figure 5.**
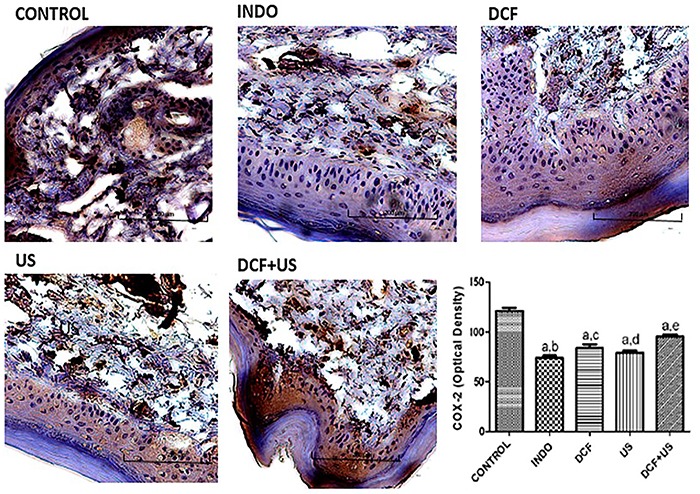
Representative photomicrographs of immunohistochemistry for cyclooxygenase-2 (COX-2) (400×, bar=200 μm) of tissues from the right paw of rats submitted to the carrageenan-induced paw edema model. The Control (untreated) group was compared with the groups treated with 20 mg/kg indomethacin, *po* (INDO), 10 mg/g diclofenac (DCF), ultrasound (US, 1 w/cm^2^, 1 min), and ultrasound associated with diclofenac (DCF+US). The graph shows the data analyzed by the ImageJ program. Data are reported as mean±SE. ^a^P<0.001 *vs* CONTROL; ^b^P<0.05 *vs* DCF; ^c^P<0.01, ^d^P<0.01 *vs* DCF+US. ^e^P<0.001 *vs* INDO. (one-way ANOVA and Newman-Keuls with *post hoc* test).

The immunohistochemical analysis revealed an abundant expression of COX-2 in the tissues from untreated paws and carrageenan-stimulated animals (Figure 5). It is possible to observe a strong brown coloration in the cytoplasm of neutrophils, eosinophils, and macrophages, indicating that COX-2 is being expressed in these cells because of the inflammatory mechanism induced by carrageenan. This expression was significantly inhibited by indomethacin, a NSAID that was used as positive control. In addition, the treatments with DCF+US (P<0.001), DCF (P<0.05), and US alone (P<0.05) significantly reduced COX-2 expression compared to the control group, suggesting that the treatments possibly interfere with the mechanisms of lipid mediator production.

In fact, phonophoresis has been reported to reduce carrageenan-induced edema, indicating that this method can affect the expression of COX-2 and thus, decrease the levels of prostaglandins. Such phenomenon can be associated with a reduction in the levels of TNF-α, because as previously demonstrated, this treatment reduced the leukocyte infiltrates, which could affect the production of this cytokine.

## Discussion

NSAIDs are widely employed in the treatment of acute and chronic pain resulting from inflammatory processes. Although these drugs present analgesic, anti-inflammatory, and antipyretic properties, they have significant side effects that can cause harm to patients. The mechanism of action of NSAIDs involves inhibition of cyclooxygenases (COX-1, COX-2, and COX-3), important enzymes in the production of lipid mediators. Thus, because COX-1 is constitutively expressed in many tissues (such as kidneys, gastric mucosa, and platelets), most of the side effects of NSAIDs are associated with the blockade of this enzyme. Therefore, the development of novel therapeutic alternatives that can be used alone or in combination with reduced doses of conventional NSAIDs can minimize the toxicity and improve treatment of inflammatory disorders (16).

In the present study, the anti-inflammatory and analgesic effects of phonophoresis alone or in association with diclofenac diethylammonium was evaluated using an experimental model of carrageenan-induced paw edema in rats. Carrageenan is a sulfated polysaccharide extracted from seaweed that has been widely used in animal models of inflammation as a tool in the screening of novel anti-inflammatory drugs (17).

An intraplantar injection of carrageenan induces an inflammatory response that is characterized by a time-dependent generation of paw edema, neutrophil infiltration, and increased levels of nitrite/nitrate and prostaglandin E2 in the exudate (18). It has been demonstrated that in this model, the edema is maximal after 3 h and remains elevated for 10 h after administration of carrageenan. Nitric oxide (NO), produced by the enzyme nitric oxide synthase (NOS), is involved in the development of inflammation shortly after administration of carrageenan, and the activation of the inducible form of this enzyme (iNOS) generates additional NO that maintains the inflammatory response. In addition, carrageenan increases the levels of many cytokines, such as TNF-α, IL-1β, and IL-6 in the inflamed paw (19). The acute vascular response triggered by carrageenan occurs in three distinct phases orchestrated by specific mediators. The first phase occurs 1 h after carrageenan administration and is characterized by an increased vascular permeability that is mediated by histamine and serotonin; the second phase develops 2 h after the challenge and is orchestrated by kinins, such as bradykinin; the third and last phase generates the most significant edema. It develops 3 h after carrageenan administration and is mediated by prostaglandins. Therefore, substances that inhibit the effects of carrageenan in this phase are thought to present anti-inflammatory properties (14).

This research demonstrated that treatment with US+DCF was more effective than treatment with US alone. The use of topical anti-inflammatory drugs such as diclofenac in the form of a gel associated with ultrasound is widely used in clinical physiotherapy, because this combination facilitates the absorption of the drug (20). However, the pharmacodynamic mechanisms underlying the effects of this combination remain to be fully elucidated. Importantly, the results obtained in this study indicated that in addition to facilitating the penetration of the drug in the tissue, the phonophoresis possibly inhibits the release of inflammatory mediators, such as prostaglandins.

The association of pentoxifylline with US+DCF produced significant and synergistic effects on edema formation, suggesting that, in addition to lipid mediators (prostaglandins), this association can affect the production of cytokines, such as TNF-α. In fact, some authors (21,22) have demonstrated that pentoxifylline inhibits the production of TNF-α by monocytes and T cells *in vitro*.

In the Hargreaves test, hyperalgesia is triggered, at least in part, by the central and peripheral release of prostaglandins in response to tissue injury. In this test, the results showed that treatment with US+DCF was effective in increasing the latency time of paw withdrawal after the stimulus, evidencing an antinociceptive effect of this association, which may be correlated with reduction of prostaglandin synthesis.

The histological and immunohistochemical analysis demonstrated that therapeutic ultrasound treatment associated with phonophoresis reduced the inflammatory cell infiltrates and the expression of COX-2. Here, it is suggested that because these treatments reduce leukocyte infiltrates, lower levels of TNF-α and other cytokines (such as IL-1β) are found in the tissue, affecting the expression of COX-2 (14). Therefore, the anti-inflammatory and antinociceptive effects of the association of US and phonophoresis may result from inhibition of COX-2 expression and reduction of prostaglandin production.

Previous studies have suggested that the synergism obtained in the association of US with DCF is due to a facilitation of transcutaneous penetration of DCF as a result of the phenomenon of cavitation and changes in the blood flow (20,23–27). However, the data obtained in this study suggest that this synergism is associated with potentiation of the anti-inflammatory mechanisms of both treatments. Clinically, these data are extremely relevant because this association could be used to reduce the dose of NSAIDs required for the treatment of inflammatory disorders, and thus, minimize their toxic effects.

The acoustic vibrations produced by therapeutic ultrasound devices induce cellular changes by altering the concentration gradient of calcium and potassium ions, affecting the transport of metabolites through the cell membrane. This phenomenon induces several tissue alterations, such as increased protein synthesis and secretion by mast cells and changes in fibroblast mobility. Therefore, alterations in the membrane permeability result in cell activation and contribute to tissue repair (10,11,28).

Therefore, it is hypothesized that the mechanism involved in the potentiation of drug absorption through the skin caused by phonophoresis is associated with the phenomenon of cavitation. Cavitation induces the formation of gaseous microbubbles in the outer layer of the skin (*stratum corneum*) that trigger microvascular hemodynamic modifications. This can lead to increased perfusion, granulation tissue formation, tissue repair in response to fibroblastic proliferation, and increase of precursor cells and disorganization of the lipids in the corneum layer. These changes increase the permeability, favoring the penetration of the drug (29–32).

In addition, this work demonstrated scientifically that the use of topical anti-inflammatory drugs in the form of gel associated with therapeutic ultrasound, which has been practiced almost empirically (especially by physiotherapy professionals), is effective.

In conclusion, the ultrasound-mediated phonophoresis alone or in association with topical diclofenac has potential for application in clinical practice, because these treatments present anti-inflammatory and analgesic effects that might be dependent on inhibition of COX-2 expression and TNF-α production.
